# Continuous glucose monitoring trajectories in patients with acute coronary syndrome

**DOI:** 10.1186/s12933-026-03169-1

**Published:** 2026-04-20

**Authors:** Arancha Díaz-Expósito, Victoria García-Ruiz, Diego Castillo-Barnes, Andrés Ortiz, Jose Ignacio Larrubia-Valle, Agustín Molinero, Fernando Puyol-Ruiz, Oscar Barquero-Alegre, Nelsa González-Aguado, Laura Martín-Chaves, Jorge Segovia-Reyes, Cristobal Urbano-Carrillo, Juan José Gómez Doblas, Manuel Jiménez-Navarro, Jorge García-Alemán, Jorge Rodríguez-Capitán, Fernando Gómez Peralta, Francesco Costa

**Affiliations:** 1 Unidad Clínica Área del Corazón, Hospital Universitario Virgen de la Victoria - IBIMA Plataforma BIONAND, CIBERCV, ISCIII, Malaga, Spain; 2 Departamento de Medicina y Dermatología, Universidad de Málaga - UMA, Málaga, Spain; 3 Communications Engineering, University of Málaga, Malaga, Spain; 4 Cardiology Department, Hospital Universitario Regional de Málaga, Malaga, Spain; 5 Endocrinology and Nutrition Unit, Hospital General de Segovia, Segovia, Spain; 6 Department of Biomedical and Dental Sciences and of Morphological and Functional Images, University of Messina, Messina, Italy

**Keywords:** Acute coronary syndrome, Continuous glucose monitoring, Glycaemic variability, Dysglycaemia, Cardiometabolic risk

## Abstract

**Background:**

Continuous glucose monitoring (CGM) captures dysglycaemia and glycaemic variability after acute coronary syndrome (ACS), but patient-level trajectories from early recovery to mid-term follow-up—particularly in people without diabetes—remain insufficiently characterized.

**Methods:**

In this prospective multicenter observational study (ORACLE program), consecutive high-risk ACS patients wore a FreeStyle Libre 3/3 Plus sensor for 14–15 days near discharge and again at ~ 4 months. We quantified CGM time-in-range metrics (70–180 mg/dL and tight range 70–140 mg/dL), time above/below range, and variability/risk indices, including within-day profiles. Clinically relevant changes were categorized using pre-specified thresholds, and predictors of worsening were explored using multivariable models.

**Results:**

Among all patients, 213 patients had analyzable baseline CGM recordings meeting quality criteria. In the early post-ACS period, median [IQR] time in range 70–180 mg/dL was 96.57% [86.91–98.92], time in tight range 70–140 mg/dL was 85.00% [62.16–93.24], time above range > 180 mg/dL was 1.12% [0.18–9.82], and time below range < 70 mg/dL was 0.43% [0.00–1.84], with a mean glucose of 114.26 mg/dL [106.26–133.96] and a median glucose management indicator of 6.04% [5.85–6.51]. CGM demonstrated marked inter-individual heterogeneity and a reproducible late-morning (10:00–12:00) vulnerability window with lower range time and higher hyperglycaemic exposure, consistent across diabetes status and similar on weekdays and weekends; adverse CGM profiles were more prominent in patients with diabetes, older individuals, and women. Although CGM parameters improved modestly during the initial monitoring period, glycaemic control showed a slight but consistent deterioration from baseline to ~ 4 months after ACS, including in patients without diabetes. Tight-range time decreased by 4.5% (*p* = 0.008) and mean glucose increased by 4.67 mg/dL (*p* = 0.03), accompanied by a parallel worsening of variability and glycaemic risk indices. In contrast, HbA1c remained stable over follow-up. Across CGM endpoints, ~ 20–40% of patients showed a worsening trajectory (20.2% by broad time-in-range thresholds); higher comorbidity burden clustered with deterioration, with hypertension and COPD independently associated with tight-range worsening.

**Conclusions:**

After ACS, CGM reveals substantial inter-individual heterogeneity and a reproducible late-morning vulnerability window. From discharge to mid-term follow-up, deterioration—also affecting patients without diabetes—may be preferentially detected by tight-range and variability/risk metrics that traditional monitoring of blood glucose and static measures such as HbA1c may overlook, supporting CGM-informed phenotyping to refine post-ACS metabolic surveillance.

**Graphical Abstract:**

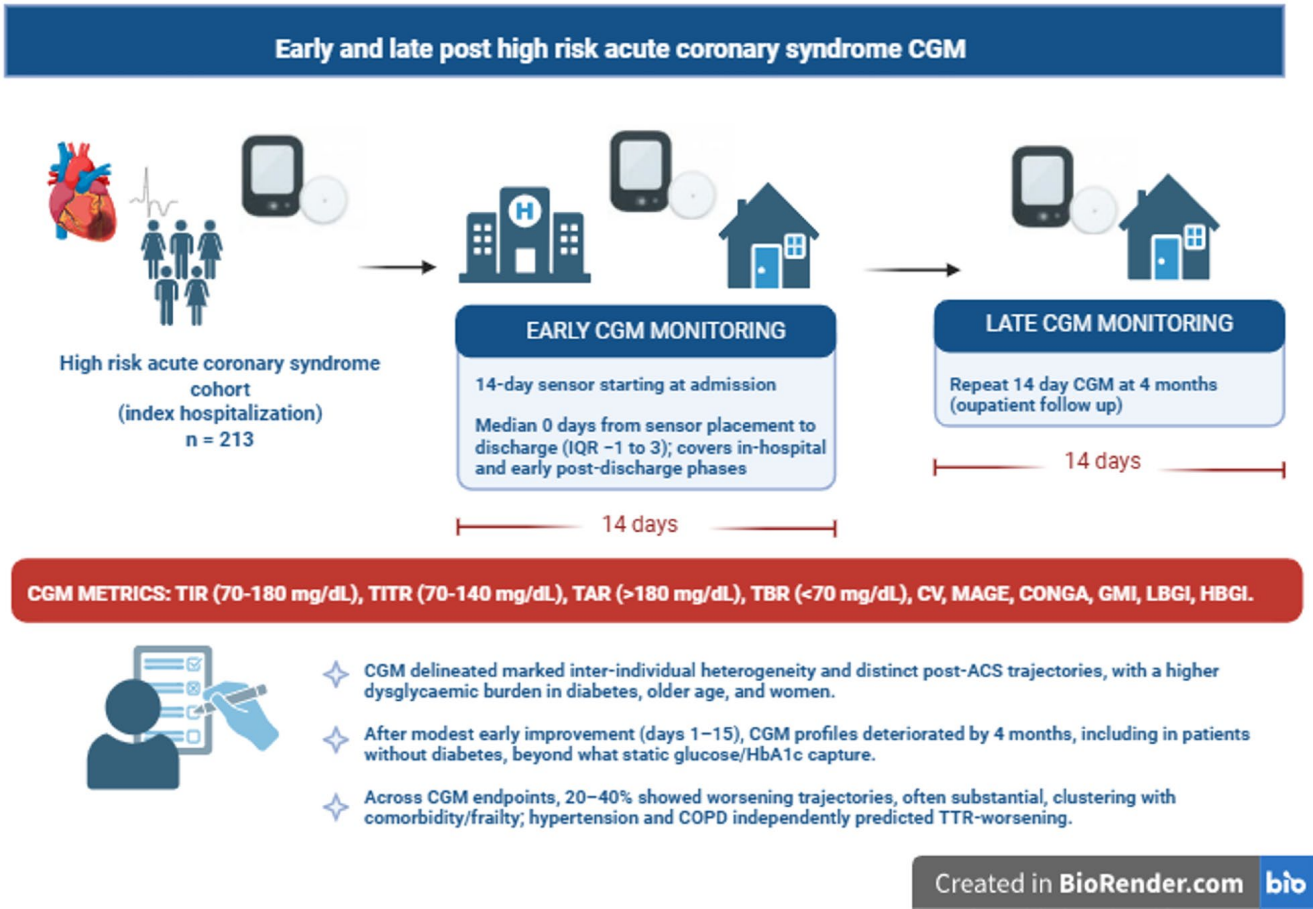

**Supplementary Information:**

The online version contains supplementary material available at 10.1186/s12933-026-03169-1.

**Research insights**

**Table:** 

What is currently known about this topic?	• Dysglycaemia and glycaemic variability after ACS contribute to residual cardiovascular risk. • CGM can uncover excursions missed by traditional glucose monitoring strategies, including in people without diabetes. • Longitudinal CGM data across early post‑ACS and follow‑up remain limited.
What is the key research question?	How do CGM-derived metrics evolve from early post‑ACS to 4 months, and which patients are prone to deterioration?
What is new?	• CGM revealed marked inter-individual heterogeneity and a late-morning (10:00–12:00) vulnerability window. • At ~ 4 months, deterioration in tight-range control/variability occurred also in patients without diabetes. • Worsening affected ~ 20–40%; clustered with multimorbidity; hypertension and COPD predicted TITR worsening.
How might this study influence clinical practice?	CGM may help identify, early after ACS, patients (including those without diabetes) who are prone to deteriorate by follow-up, enabling more tailored metabolic surveillance and follow-up intensity beyond what static glucose or HbA1c can capture.

## Background

Acute coronary syndromes (ACS) arise within a complex metabolic milieu, in which diabetes mellitus is a major determinant of risk through both its direct acceleration of atherosclerotic vascular disease and its indirect effects mediated by hyperglycaemia and glycaemic variability (GV). These dynamic disturbances may modulate inflammation, endothelial dysfunction, and thrombogenicity, thereby contributing to the residual risk that persists after myocardial infarction (MI) [1–3]. Accordingly, reducing the risk of recurrent ischaemic events may require a higher level of awareness and more proactive, individualized management of residual glycaemic risk in this population. Traditional biomarkers—principally single-point glucose and glycated haemoglobin (HbA1c)—reflect average glycaemia over time but fail to capture short-term fluctuations that could influence clinical outcomes and long-term prognosis [4]. Continuous glucose monitoring (CGM) enables high-resolution assessment of glucose dynamics, providing quantitative indices of variability, hypoglycemic and hyperglycemic exposure, and overall metabolic stability [5]. In stable coronary artery disease, observational studies have linked CGM-derived metrics—including time in range (TIR), time above range (TAR), and GV indices—with overall atherosclerotic burden and high-risk plaque features on coronary imaging, suggesting that ambulatory dysglycaemia may influence plaque progression and vulnerability [6–8].

Recent consensus statements support complementing static markers such as HbA1c with pattern-based CGM metrics to enhance mechanistic insight and refine risk stratification and therapeutic targeting [9]. Moreover, CGM has proven feasible and informative for metabolic profiling in individuals without diabetes, although robust cardiovascular outcome data remain scarce [10]. Evidence on CGM-derived metrics during the acute and early recovery phases after ACS remains limited, particularly in unselected, high-risk populations that include patients with and without diabetes. Characterizing CGM trajectories during this vulnerable period may provide valuable insights into metabolic signatures and mechanisms underlying residual cardiovascular risk. In the current study we sought to systematically characterize glycemic profiles using CGM early after ACS and at mid-term follow-up in order to: (i) describe the overall and subgroup patterns of glucose control after ACS, (ii) evaluate temporal changes in CGM-derived metrics between discharge and follow-up, and (iii) identify clinical predictors of unfavorable metabolic trajectories.

## Methods

### Study population and design

This is a pre-specified descriptive study of the Optimize Risk Prediction after Myocardial Infarction through Artificial Intelligence and Multidimensional Evaluation (ORACLE) program, a prospective, observational, multicenter study which consecutively enrolls patients with high-risk ACS. High-risk ACS patients were eligible if at least two of the following criteria were present: age > 65 years; diabetes mellitus; multivessel coronary artery disease; peripheral artery disease; chronic kidney disease; prior stroke (at any time) or prior transient ischaemic attack within the last 6 months; prior myocardial infarction; complex percutaneous coronary intervention; prior PCI or coronary artery bypass grafting; heart failure; body mass index > 27 kg/m²; anticipated long-term use of an oral anticoagulant; haemoglobin < 11 g/dL; spontaneous bleeding requiring hospitalization or transfusion in the past 12 months; bleeding diathesis; active malignancy; or previous spontaneous intracranial haemorrhage. Exclusion criteria were intentionally limited to allow a contemporary all-comer population and included: age < 18 years; low life expectancy (< 1 year) at the treating physician’s discretion; pregnancy or breastfeeding; and non-significant coronary artery disease on angiography (< 30% stenosis in the left main stem or < 50% in other coronary segments). Patients were enrolled after the index percutaneous revascularization, once clinical stabilization had been achieved and in close proximity to hospital discharge.

CGM was initiated at study inclusion and repeated at the 4-month follow-up using a dedicated wearable sensor (FreeStyle Libre 3 and Libre 3 Plus; Abbott Diabetes Care) worn for 14–15 consecutive days. Sensor data were automatically transmitted in a blinded fashion to a dedicated reader kept at the patient’s home. After each recording period, the reader was returned to the study team and the sensor was discarded. CGM was performed in all eligible participants, including those with and without a prior diagnosis of diabetes mellitus, provided they met the study’s high-risk ACS inclusion criteria. There was no requirement for prior CGM use; instead, CGM was systematically provided as part of the study protocol. Other clinical, demographic and biochemical data were collected at baseline and follow-up visits, according to the study protocol. The study has been approved by the local Institutional Review Board, and an Informed Consent Form was signed by the patient or the legal guardian before study inclusion. The study was conducted according to the ethical principles of the Declaration of Helsinki and the protocol was prospectively registered in a public repository (ClinicalTrials.gov—NCT06993415).

### Preprocessing and feature analysis

Among all patients included in the study (*N* = 273) only patient who reached prespecified CGM quality criteria have been included in the final cohort. CGM time-series downloaded from the CGM device were evaluated to ensure a recording duration between 5 and 15 days and to exclude series with extended measurement gaps (> 3 h). The days immediately preceding and following each large gap were removed to maintain signal continuity. Temporal features were derived according to the international CGM consensus metrics defined by Battelino et al. [5], including: time in range (TIR, 70–180 mg/dL − 3.9–10 mmol/L), time in tight range (TITR, 70–140 mg/dL- 3.9–7.8 mmol/L), time above range (TAR, > 180 mg/dL - >10 mmol/L), time below range (TBR, < 70 mg/dL - <3.9 mmol/L), mean amplitude of glycaemic excursions (MAGE), coefficient of variation (CV) and standard deviation (SD) of glucose, Glucose Management Indicator (GMI), Glycaemic Risk Index (GRI), low blood glucose index (LBGI), high blood glucose index (HBGI), and continuous overall net glycaemic action over 24 h (CONGA-24). These indices, computed from validated CGM traces at each time point (early post-ACS and ~ 4-month follow-up), served as quantitative variables for subsequent cross-sectional and longitudinal analyses.

### Statistical analysis

For each extracted feature, the distributional normality was assessed using the Anderson–Darling test. Normally distributed variables were summarized as mean ± standard deviation, whereas non-normal variables were expressed as the median with the interquartile range. When the sample sizes were small (< 8 observations per group), non- parametric procedures were applied to avoid unreliable parametric estimates. Between-group comparisons were performed using the Student’s t-test for normally distributed data and the Mann–Whitney U test otherwise. Paired comparisons (i.e. baseline versus follow-up) used paired t-tests or Wilcoxon signed-rank tests. For multi-group analyses, one-way analysis of variance (ANOVA) was performed under normality, whereas the Kruskal–Wallis test was used otherwise. If an overall difference was detected (*p* < 0.05), pairwise comparisons were adjusted for multiple testing using Tukey’s correction for parametric data or Dunn–Bonferroni correction for non-parametric data. To examine the combined influence of CGM-derived metrics and clinical covariates, multivariate analyses were performed after assessing the potential collinearity among the predictors. Multiple linear regression was used when the dependent variable was continuous and logistic regression was used when the outcome was binary. When several comparisons were performed, Bonferroni or Benjamini–Hochberg corrections were performed depending on the test dependency. All analyses were conducted using Python 3.12 and RStudio (version 2024.09.0, Build 375) with R (version 4.4.1).

## Results

### Baseline clinical characteristics

A total of 213 consecutive patients with high-risk ACS were included in the current analysis. Mean age was 66.3 ± 11.6 years and 77.9% were male. Cardiovascular comorbidity burden was substantial, in line with the high-risk cohort included (Supplementary Table 1). Most patients presented with either ST-segment elevated MI or non-ST segment elevated MI (84.9%). Patients with an established diagnosis of diabetes mellitus were 44.1% (0.9% type 1 and 43.6% type 2). All patients with type 1 diabetes were treated with insulin, whereas type 2 diabetes were treated with contemporary drug combinations (Supplementary Table 1).

### Baseline CGM metrics in the total cohort

**Fig. 1:**
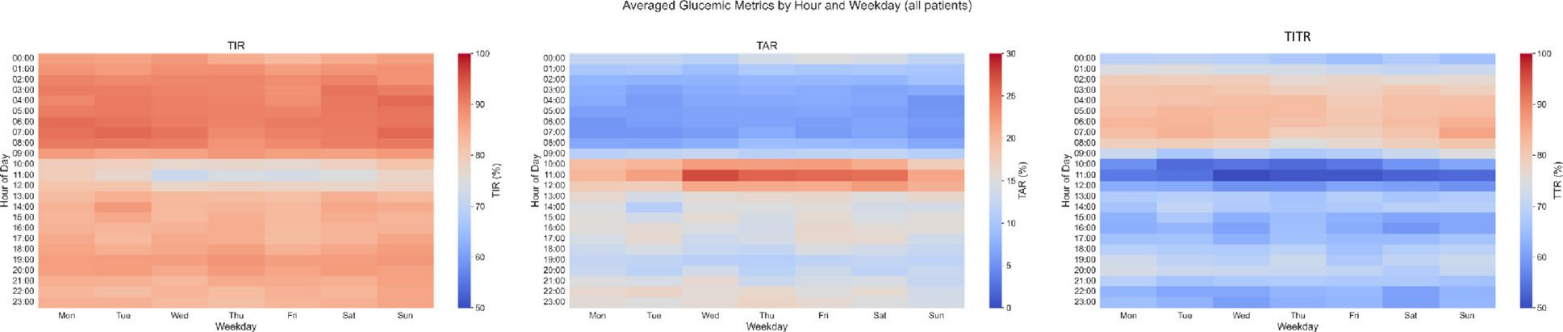
Heatmap presenting averaged time-in-range (**A**), time-above range (**B**) and time-in tight range (**C**) within daily hours and weekdays at baseline. Red color represent higher, while blue color represent lower % of time in and above range. TAR: Time above range; TIR: Time in range; TITR: Time in tight range

**Fig. 2:**
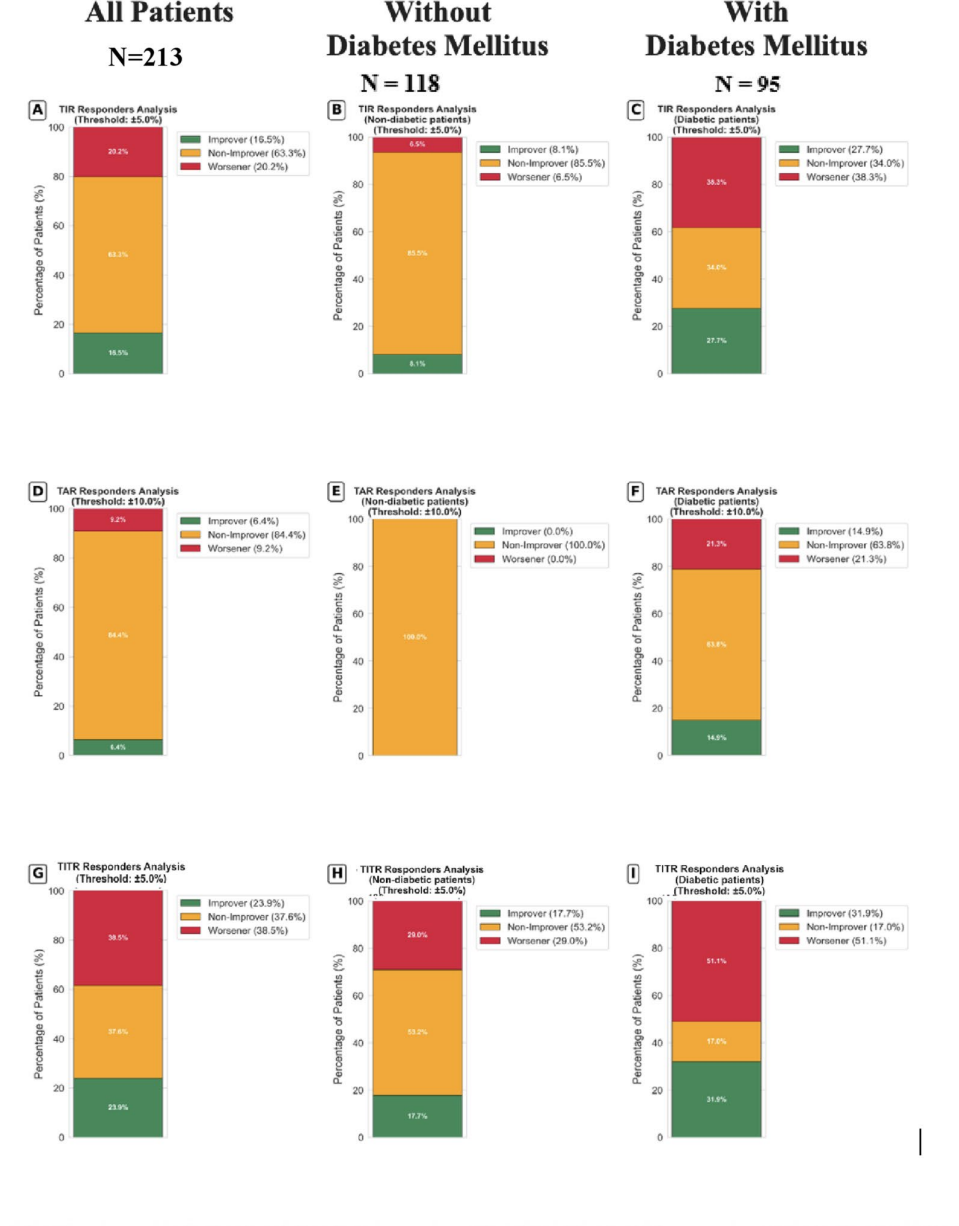
CGM metric trajectories between baseline and 4-months follow-up in patients early after an acute coronary syndrome. Metrics for TIR (**A**–**C**), TAR (**D**–**F**) and TITR (**G**–**I**) are presented in the overall patient population, in non-diabetic and diabetic patients. Improvers, non-improvers and worseners are defined as a ΔTIR ≥ + 5%, within ± 5% and ≤ − 5%, ΔTAR ≤ − 10%, within ± 10% and ≥ 10%, and Δ TITR ≥ + 5%, within ± 5% and ≤ − 5%, respectively. TAR: Time above range; TIR: Time in range; TITR: Time in tight range

The median interval between CGM device placement and hospital discharge was 0 days (IQR: −1 to 3). Median [IQR] TIR was 96.57 [86.91–98.92], TITR was 85.00 [62.16–93.24], while TAR and TBR were 1.12 [0.18–9.82] and 0.43 [0.00–1.84], respectively (Table 1). Mean glucose was 114.26 [106.26–133.96], corresponding to a GMI of 6.04 [5.85–6.51]. The least favorable within-day CGM profile occurred between 10:00 and 12:00, irrespectively of diabetes status (Fig. 1, Suplementary Figs. 1 and 2). Hourly heatmaps showed a nadir in TIR, mirrored by a peak in TAR during this late-morning window, without a compensatory increase in hypoglycaemia indices afterwards. This pattern was observed on both weekdays and weekends and was not driven by an isolated outlier hour. When baseline CGM metrics within the first 3 days after study inclusion (near in-hospital phase—day 1–3) were compared to the last 3 days of recording (out-of-hospital phase—day 13–15) glycaemic control improved modestly. TIR increased from 93.28 [76.28–98.08] to 96.58 [85.57–99.70] (*p* = 0.001), with a similar increase in the TITR and a reduction in TAR, with a parallel improvement in variability metrics (Table 1). CGM metrics at baseline among relevant subgroups are presented in Table 1. Patients with diabetes mellitus exhibited markedly worse CGM metrics, compared to patients without diabetes, with substantially lower TIR (98.63 [95.82–99.40] vs. 85.29 [60.07–93.30]) and TITR (89.25 [82.47–94.53] vs. 57.46 [25.94–73.67]), higher TAR (89.25 [82.47–94.53] vs. 57.46 [25.94–73.67]) and mean glucose (111.11 [105.09–118.79] vs. 139.85 [125.77–170.81]).

Among type 2 diabetes treatment class, insulin-treated profiles showed the most adverse pattern. Among patients not treated with insulin, there was no difference in CGM metrics across patients assigned to one, two and > 2 non-insulin diabetes medical treatments. Women and elderly patients presented worse CGM metrics, while no difference was observed among patients presenting with or without MI, those with lower LVEF or BMI categories.

**Table 1:** Baseline CGM-derived metrics early after an acute coronary syndrome in the total cohort and prespecified subgroups

	Total cohort (*n* = 213)	Without Diabetes Mellitus (*n* = 116)	With Diabetes Mellitus (*n* = 97)	*P*	Men (*n* = 165)	Women (*n* = 48)	*P*	First 3 days (*n* = 211)	Last 3 days (*n* = 211)	*P*
Time in range 70–180, %	96.57 [86.91–98.92]	98.63 [95.82–99.40]	85.29 [60.07–93.30]	< 0.001	96.06 [87.00–98.94]	89.54 [79.76–96.43]	0.007	93.28 [76.28–98.08]	96.58 [85.57–99.70]	0.001
Time in range 70–140, %	85.00 [62.16–93.24]	89.25 [82.47–94.53]	57.46 [25.94–73.67]	< 0.001	82.24 [62.16–92.53]	71.15 [43.24–88.83]	0.037	76.12 [47.62–89.76]	80.31 [62.72–92.12]	0.014
Time above range > 180, %	1.12 [0.18–9.82]	0.45 [0.00–1.37]	14.02 [5.17–39.79]	< 0.001	1.48 [0.18–10.79]	8.33 [0.47–19.68]	0.028	2.67 [0.00–19.78]	1.34 [0.00–11.20]	0.059
Time below range < 70, %	0.43 [0.00–1.84]	0.47 [0.05–1.81]	0.11 [0.00–1.10]	0.008	0.17 [0.00–1.11]	0.43 [0.02–2.39]	0.220	0.00 [0.00–1.91]	0.00 [0.00–0.51]	0.113
Time < 54, %	0.00 [0.00–0.20]	0.00 [0.00–0.20]	0.00 [0.00–0.11]	0.088	0.00 [0.00–0.15]	0.00 [0.00–0.21]	0.838	0.00 [0.00–0.00]	0.00 [0.00–0.00]	0.840
Time ≥ 250, %	0.00 [0.00–0.56]	0.00 [0.00–0.00]	0.82 [0.00–7.73]	< 0.001	0.00 [0.00–0.53]	0.34 [0.00–2.51]	0.003	0.00 [0.00–1.33]	0.00 [0.00–0.00]	0.062
Mean glucose, mg/dL	114.26 [106.26–133.96]	111.11 [105.09–118.79]	139.85 [125.77–170.81]	< 0.001	117.04 [108.62–136.83]	130.37 [111.11–150.27]	0.102	121.82 [108.83–149.51]	118.88 [108.12–136.58]	0.185
Coefficient of variation, %	23.55 [17.24–34.40]	19.70 [16.22–24.01]	35.43 [27.17–46.17]	< 0.001	23.78 [18.37–34.23]	32.76 [21.25–42.08]	0.011	26.20 [19.81–36.45]	22.65 [16.93–30.61]	0.001
Standard deviation, mg/dL	19.90 [16.64–25.42]	17.82 [14.99–21.38]	23.94 [21.00–27.44]	< 0.001	19.86 [16.90–24.38]	23.13 [19.44–27.05]	0.005	20.85 [17.20–24.88]	18.52 [15.16–22.78]	< 0.001
Glucose management indicator, %	17.90 [14.83–20.77]	16.08 [13.76–19.20]	20.28 [17.67–23.74]	< 0.001	17.82 [15.22–21.14]	20.31 [15.83–24.12]	0.007	19.13 [15.81–22.78]	16.54 [14.06–20.58]	< 0.001
MAGE, mg/dL	6.04 [5.85–6.51]	5.97 [5.82–6.15]	6.66 [6.32–7.40]	< 0.001	6.11 [5.91–6.58]	6.43 [5.97–6.90]	0.102	6.22 [5.91–6.89]	6.15 [5.90–6.58]	0.185
LBGI	47.62 [34.97–65.31]	38.59 [32.60–47.70]	67.16 [54.99–84.76]	< 0.001	47.63 [35.98–65.06]	61.68 [41.57–76.54]	0.021	51.74 [38.75–71.72]	45.11 [34.13–60.88]	0.007
HBGI	0.57 [0.17–1.00]	0.59 [0.32–1.10]	0.17 [0.04–0.66]	< 0.001	0.42 [0.14–0.92]	0.40 [0.14–0.91]	0.933	0.37 [0.07–1.10]	0.29 [0.08–0.77]	0.419
CONGA-24, mg/dL	0.74 [0.32–2.38]	0.42 [0.22–0.89]	3.15 [1.62–8.02]	< 0.001	0.91 [0.34–2.61]	1.93 [0.49–4.22]	0.044	1.16 [0.41–4.15]	0.95 [0.33–2.56]	0.046
Hypoglycaemic events, n	21.97 [16.13–30.19]	18.27 [14.66–22.95]	30.75 [24.47–37.55]	< 0.001	22.63 [16.64–29.96]	29.48 [20.69–35.12]	0.007	23.34 [17.98–31.61]	21.56 [16.19–30.45]	0.137
Hyperglycaemic events, n	1.00 [0.00–6.00]	2.00 [0.00–5.00]	1.00 [0.00–3.00]	0.029	1.00 [0.00–5.00]	1.00 [0.00–5.25]	0.313	0.00 [0.00–2.00]	0.00 [0.00–1.00]	0.045

All variables are presented as median [IQR], as all were classified as non-normally distributed according to the Anderson–Darling normality test. DM, diabetes mellitus. MAGE, mean amplitude of glycaemic excursions; LBGI, low blood glucose index; HBGI, high blood glucose index; CONGA-24, continuous overall net glycaemic action over 24 h; LVEF, left ventricular ejectionfraction; MI, myocardial infarction.First 3 days” and “Last 3 days” refer to the initial and final 3-day CGM windows, respectively. These windows are anchored to the CGM recording (rather than hospitalisation status) because length of stay and the proportion of CGM worn in-hospital vs. after discharge varied across individuals

### CGM at 4-month follow-up and patients’ trajectories

Between baseline and 4-month follow-up after the index ACS admission, there was a slight but consistent worsening of CGM metrics (Table 2), both in patients with and without diabetes (Supplementary Table 2). TITR decreased by 4.5% (*p* = 0.008) and mean glucose increased by 4.67 mg/dL (*p* = 0.03), with concordant rises multiple variability indexes. Among patients without diabetes, there was a significant 3% reduction in the TITR (*p* = 0.03) and an increase in hyperglycemia events between baseline and 4 months, with a consistent significant increase in most variability and risk indices (SD increased by 1.18 mg/dL *p* = 0.043, MAGE by 2.84 mg/dL *p* = 0.03, HBGI by 0.14 *p* = 0.03 and CONGA-24 by 1.12 mg/dL *p* = 0.02). In contrast, no consistent changes were observed in HbA1c in the total cohort, which remained essentially unchanged between baseline and 4-month follow-up (6.10 [5.60–6.93] vs. 6.10 [5.80–7.00]; *p* = 0.24) *)*, irrespectively to diabetes status (diabetes mellitus 6.50 [5.70–7.20] vs. 6.40 [5.90–7.35]; *p* = 0.52; without diabetes mellitus 6.00 [5.60–6.60] vs. 6.00 [5.70–6.60]; *p* = 0.31. We categorized relevant changes in TIR, defining groups of patients identified as improvers (ΔTIR ≥ + 5%), non-improvers (ΔTIR = ± 5%) and worseners (ΔTIR ≤ − 5%). Using these thresholds, 16.5% of patients were identified improvers, 63.3% non-improvers and 20.2% worseners (Fig. 2). When exploring the subgroups of patients with and without diabetes mellitus, worseners according to TITR metrics were 51.1% and 29% respectively (Fig. 2). No difference in baseline CGM metrics was observed among patients with a worsening trajectory (Supplementary Table 3).

**Table 2:** CGM metrics change between baseline and 4-months follow-up

Metric	Baseline (median [IQR],)	Second visit (median [IQR],)	*p*
TIR 70–180 (%)	96.57 [86.91–98.92]	96.24 [81.15–98.53]	0.260
Time 70–140 (%)	85.00 [62.16–93.24]	80.48 [48.65–90.94]	0.008
TAR > 180 (%)	1.12 [0.18–9.82]	1.69 [0.27–12.52]	0.068
TBR < 70 (%)	0.43 [0.00–1.84]	0.32 [0.00–1.70]	0.295
TBR level 2 (< 54, %)	0.00 [0.00–0.20]	0.00 [0.00–0.07]	0.370
TAR level 2 (≥ 250, %)	0.00 [0.00–0.56]	0.00 [0.00–0.57]	0.623
Mean glucose (mg/dL)	114.26 [106.26–133.96]	118.93 [108.03–146.22]	0.035
CV (%)	19.90 [16.64–25.42]	20.39 [17.40–24.78]	0.250
GMI (%)	6.04 [5.85–6.51]	6.15 [5.89–6.81]	0.035
MAGE (mg/dL)	47.62 [34.97–65.31]	48.67 [37.78–68.99]	0.011
LBGI	0.57 [0.17–1.00]	0.38 [0.11–1.05]	0.212
HBGI	0.74 [0.32–2.38]	0.96 [0.39–3.28]	0.041
CONGA-24 (mg/dL)	21.97 [16.13–30.19]	23.30 [17.38–32.90]	0.015
Hypoglycaemia events (n)	1.00 [0.00–6.00]	1.00 [0.00–6.00]	0.454
Hyperglycaemia events (n)	4.00 [1.00–18.00]	8.00 [1.00–24.00]	0.004

All variables are presented as median [IQR], as all were classified as non-normally distributed according to the Anderson–Darling normality test

CONGA-24, Continuous Overall Net Glycemic Action over 24 h; CV, Coefficient of Variation; GMI, Glucose Management Indicator; HBGI, High Blood Glucose Index; LBGI, Low Blood Glucose Index; MAGE, Mean Amplitude of Glycaemic Excursions; SD, Standard Deviation; TAR, Time Above Range; TBR, Time Below Range

**Fig. 3:**
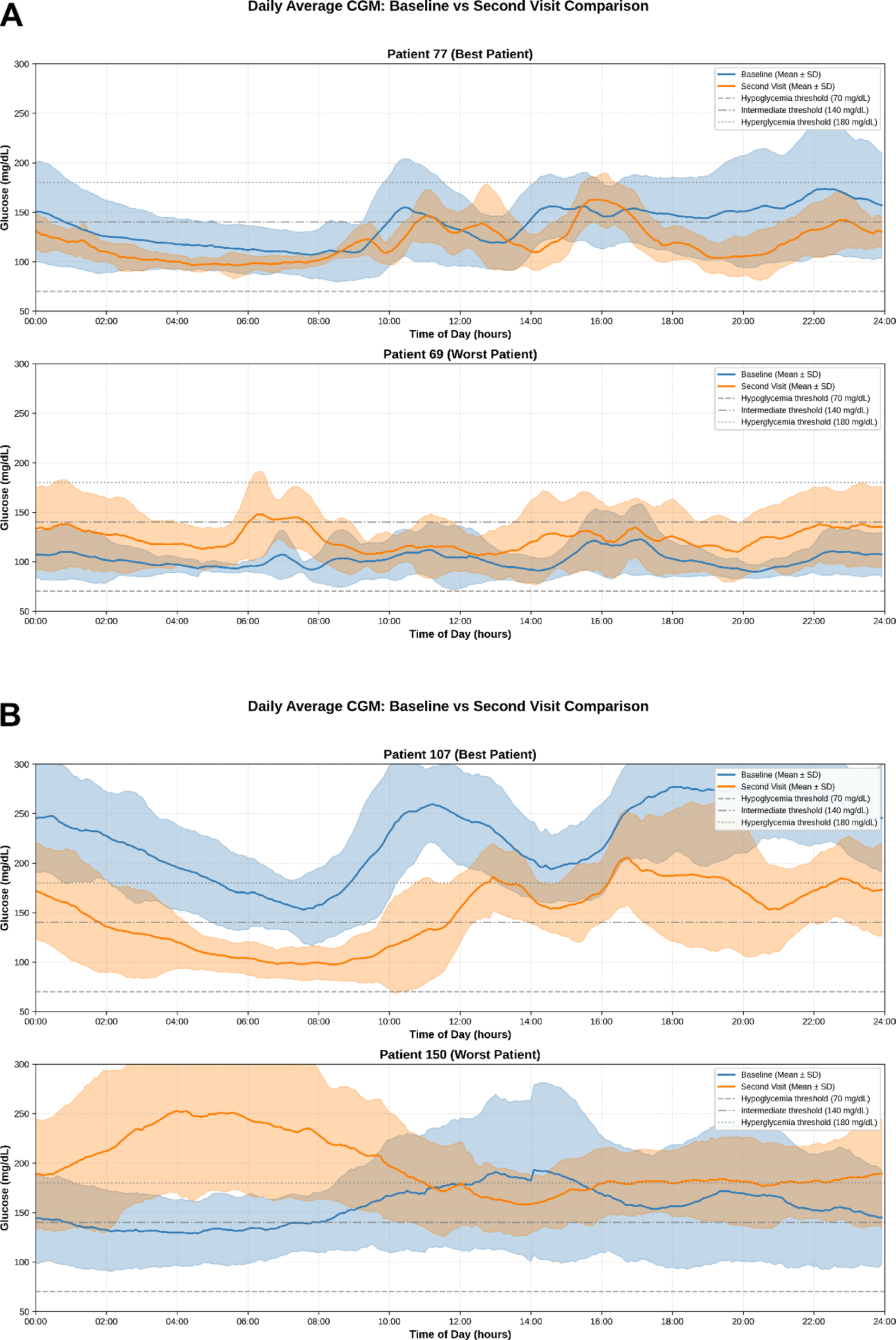
Heterogeneity in single patient trajectories between baseline and 4-month follow-up. Patients with the best (upper panel) and worst (lower panel) CGM trajectory are presented among patients without diabetes mellitus (**A**) and with diabetes mellitus (**B**)

Individual patient trajectories were heterogeneous, with substantial improvements and deteriorations coexisting despite relatively stable cohort-level averages. Notably, extremes of response were evident within our cohort both in patients with and without diabetes mellitus (Fig. 3). Among patients without diabetes, the top improver (Patient 77), TITR rose from 72.1% to 81.2% (Δ + 9.1%), whereas the greatest deterioration (Patient 69) declined from 86% to 65.4% (Δ − 20.6%), while among patients with diabetes the top improver (Patient 107) rose from 4.6% to 51.8% (Δ + 47.2%) whereas the greatest deterioration (Patient 150) declined from 53.2% to 12.3% (Δ − 40.9%) (Fig. 3). No significant medical treatment changes between baseline and follow-up were implemented (i.e. one patient was added SGLT2i and one fewer patient received insulin at 4-months follow-up; both patients belonged to the non-improvers group).

**Fig. 4:**
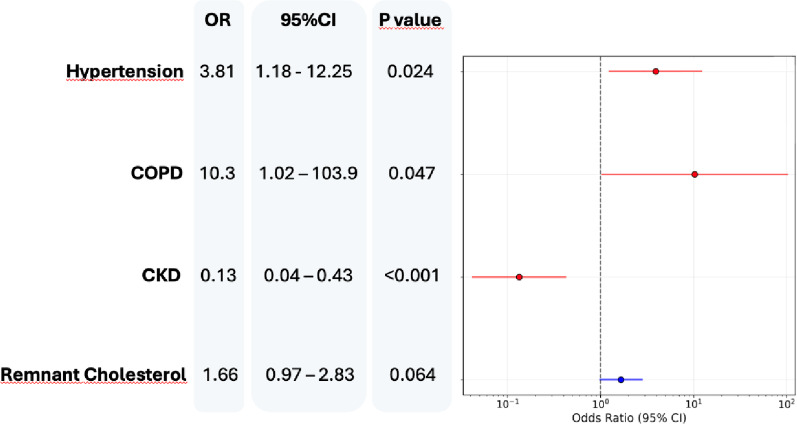
Multivariable model for clinical predictors of time-in-tight-range worsening trajectories between baseline and 4-months follow-up. Worseners were defined as a Δ TITR ≤ − 5%. *CI* Confidence Interval, *CKD* Chronic Kidney Disease, *COPD* Chronic Obstructive Pulmonary Disease, *OR* Odds Ratio

Clinical and glucometric predictors for worsening according to different CGM metrics were explored. Univariate predictors of worsening according to TIR were COPD (OR 6.22, 95% CI 1.28–30.24; *p* = 0.023), frailty (OR 13.58, 1.34–137.78; *p* = 0.027), higher discharge glucose (OR 1.85, 1.19–2.89; *p* = 0.006) HbA1c (OR 1.67, 1.06–2.63; *p* = 0.028), creatinine (OR 1.62, 1.04–2.52; *p* = 0.033) and urea (OR 1.59, 1.01–2.49; *p* = 0.044), together with increased carotid IMT (OR 1.66, 1.06–2.59; *p* = 0.027). Consistent findings were observed also for TAR- and TITR -defined worseners. In a multivariable model, hypertension (OR = 3.81; *p* = 0.024) and COPD (OR = 10.3; *p* = 0.047) were independently associated with higher odds of worsening TITR, whereas renal disease was associated with lower odds (Fig. 4). Multivariable models for TIR- or TAR-worsening were not feasible because of the limited number of patients among categories. Baseline variability glucometric indices (i.e. CONGA-24, MAGE, CV intraday and CV) were not associated to a worsening TITR status at univariate analysis, neither when included in a multivariable model of both clinical and glucometric features (Supplementary Fig. 3).

## Discussion

The main findings of the current study presenting baseline and follow-up CGM data from a prospective cohort of high-risk ACS patients can be summarized as follows:

1. CGM revealed marked inter-individual heterogeneity, and distinct patient-level trajectories among patients with recent ACS. Glycemic control was poorer during the late morning hours, without significant differences between weekdays and weekends. Worse CGM metrics were consistently observed in patients with diabetes, in older individuals, and in women.
2. Although a modest improvement in CGM parameters was observed during the first 15 days after the index event, overall glycemic control deteriorated from baseline to follow-up, importantly, this deterioration was also visible in patients without diabetes, indicating a spectrum of post-ACS metabolic worsening that may be overlooked by static glucose and HbA1c.
3. Between 20% and 40% of patients experienced a worsening glycemic trajectory from hospitalization to follow-up across different CGM metrics. Higher comorbidity burden was associated with an unfavorable trajectory, with hypertension and COPD emerging as independent predictors of TITR worsening in multivariable analysis.

This is the first study to evaluate detailed CGM-based trajectories among patients with high-risk ACS, including individuals with and without diabetes mellitus. Residual glycaemic risk after ACS is an important—and often under-recognized—component of overall residual cardiovascular risk. In this context, the key novelty of our study is the description of patient-level CGM trajectories after ACS, demonstrating that dysglycaemia evolves heterogeneously in this population. By capturing variability and excursions beyond what is reflected by intermittent capillary testing or single time-point laboratory values, CGM provides a practical approach to phenotype post-ACS dysglycaemia and to potentially provide a more precise monitor of patient-level risk over time. In this context, we identified a reproducible within-day feature: a 10:00–12:00 “hotspot” of poorer glycemic control, which was stable across weekdays and weekends. This pattern is in line with prior ACS CGM evidence [11] and is biologically plausible, given experimental data showing adverse effects of circadian phase on glucose tolerance [12], and real-world CGM findings of larger postprandial excursions when identical snacks are consumed later [13]. Whether, an adjustment in medical treatment timing could improve glucometric control is a hypothesis that may merit testing.

Importantly, we observed for the first time that a substantial proportion of patients with ACS exhibited a worsening glycaemic trajectory during follow-up. The two-phase pattern—modest improvement over the first 15 days followed by deterioration at 4 months—highlights the dynamic nature of post-ACS glycaemic control. The early improvement is plausibly driven by acute-phase resolution (including rebalancing of counter-regulatory hormones as stress subsides), together with treatment optimization and heightened early post-event adherence. In contrast, the subsequent “drift” at 4 months may reflect attenuation of these initial effects and progressive worsening of metabolic balance.

In addition, individual-level profiling revealed marked glucose fluctuations in selected patients, irrespective of diabetes status—findings that may be clinically relevant when considering residual glycaemic risk in this high ischaemic-risk population. Prior ACS CGM studies also suggest that the absence of diabetes does not equate to metabolic stability. In the cohort reported by Radermecker et al., clinically relevant hyperglycemic episodes (> 140 mg/dl − 7.8 mmol/L) were captured by CGM in 17 of 21 patients without known diabetes, whereas routine capillary testing detected elevated values in only 7 [11]. Similarly, another series found a high prevalence of abnormal day-to-day variability (MODD) reaching 68%, despite only 32% of patients having a prior diagnosis of diabetes [14]. Taken together, these data support the concept that many patients labelled as non-diabetic already sit on a spectrum of dysglycaemia and glycaemic instability early after ACS—features that conventional spot measurements and HbA1c can underestimate. Whether additional treatment based on these dynamic metrics could further reduce risk of recurrent ischemic events after an ACS remains speculative. Notwithstanding, this novel framing aligns with a broader shift in cardiometabolic therapeutics, where treatment benefit is increasingly being demonstrated beyond a strict diabetes/no-diabetes dichotomy. In the SELECT trial, among overweight patients with established cardiovascular disease (mostly MI) without diabetes mellitus, semaglutide significantly reduced major cardiovascular events (MACE) [15]. In DAPA-MI, dapagliflozin in MI patients without diabetes was associated with favourable cardiometabolic effects without a reduction in MACE [16]; notably, dapagliflozin attenuated metabolic deterioration, reducing incident type 2 diabetes both in patients with impaired glycaemic control within the prediabetes range and in those with baseline normoglycaemia, among whom progression to diabetes was reduced by 60% [17]. Collectively, these data support the potential value of modern metabolic therapies across the dysglycaemia spectrum after MI.

In this context, CGM may provide a more precise means of positioning patients along the dysglycemia spectrum and of tracking their metabolic trajectories, thereby helping to identify those who may benefit from intensified metabolic management. Ongoing studies, including GLAM-study (NCT05431296) [18], further underscore the growing interest in prolonged post-ACS CGM monitoring.

Building on the trajectory framework described above, the finding that approximately one-fifth to two-fifths of patients worsened across multiple CGM metrics—and that this deterioration clustered among those with greater comorbidity and frailty—suggests that post-ACS dysglycaemic trajectories are not randomly distributed, but concentrate within a particularly vulnerable clinical phenotype. In our cohort, univariate analyses linked worsening trajectories to markers of frailty and vasculo-metabolic impairment, while multivariable modelling identified hypertension and COPD as independent predictors of TITR worsening. These conditions may reflect convergent pathways of metabolic vulnerability—insulin resistance/metabolic syndrome, chronic low-grade inflammation, reduced physical activity, and, in COPD, intermittent systemic corticosteroid exposure—thereby amplifying post-discharge stressors and limiting tight-range glycaemic control. Consistently, patients with diabetes and older individuals showed lower TIR, higher TAR, and greater glycaemic variability, indicating a higher metabolic burden that CGM can quantify and that may help prioritize these groups for closer post-ACS metabolic follow-up. Women also tended to exhibit more adverse CGM profiles; however, this signal largely paralleled their older age and higher comorbidity burden, suggesting risk clustering rather than sex-specific biology per se, consistent with evidence that women with ACS often present later, with more comorbidities and a more complex risk profile, and may receive less individually tailored secondary prevention in routine care [19–21].Overall, these data support the concept that CGM can help identify multimorbid and frail patients as a priority group for closer metabolic surveillance and proactive therapeutic adjustment after ACS. If a more stringent CGM-based strategy is pursued, such patients with the highest-risk may be the most appropriate candidates for intensified monitoring, in line with accumulating evidence that CGM-derived glycaemic variability—particularly indices such as MAGE—is independently associated with short- and long-term outcomes after ACS [22–25].

This study has several limitations. First, as an observational study, it cannot support causal inferences between CGM patterns and clinical outcomes. Although 274 patients were enrolled, only 213 CGM recordings met strict prespecified quality criteria; while this strengthens internal validity, it may under-represent more clinically unstable or less adherent patients. The sample size, although substantial for intensive CGM phenotyping, remains limited for multivariable modelling and subgroup analyses; therefore, estimates of TIR/TAR/TITR trajectory groups and their predictors should be regarded as hypothesis-generating. The ± 5% thresholds used to define “improvers” and “worseners,” while clinically plausible and consistent with prior work [26], are inherently arbitrary and warrant prospective validation against outcomes. These findings should also be interpreted cautiously given the observational design and the absence of a control group. In addition, CGM was initiated in close proximity to discharge and early analyses relied on short 3-day windows, which may be sensitive to day-to-day variability and to in-hospital factors that were not systematically captured. Meal timing and medication timing were not recorded, so exploratory time-of-day patterns should be considered hypothesis-generating. After the baseline monitoring period, usual care was continued and the use of personal CGM devices (particularly among patients with diabetes) was not specifically controlled; importantly, the study CGM was used for data collection and was not intended to guide therapeutic decisions. Finally, the cohort was predominantly male and background diabetes therapy patterns (including relatively high SGLT-2 inhibitor use and lower GLP-1 receptor agonist use) may have influenced CGM-derived profiles and limit generalizability. Larger ACS cohorts with longer CGM monitoring and follow-up are needed to confirm the robustness of these findings and their relationship with clinical outcomes. Finally, the study was not powered for hard clinical endpoints, and whether these CGM phenotypes translate into differences in recurrent events (e.g., reinfarction, heart failure hospitalization, or mortality) remains to be established.

## Conclusions

In this prospective multicentre cohort of high-risk ACS patients, CGM revealed substantial inter-individual heterogeneity and a dynamic post-event glycaemic course, with modest early improvement followed by deterioration at 4 months. Twenty to forty% of patients exhibited worsening trajectories that clustered in those with a higher comorbidity burden, both with and without diabetes—patterns unlikely to be captured by static glucose or HbA1c assessments. These findings support CGM-based follow-up as a promising strategy to monitor residual glycaemic risk and to inform future trials testing intensified, CGM-guided optimisation of metabolic therapy to improve glucometric control and potentially reduce cardiovascular events in patients at high ischaemic risk.

## Supplementary Information

Supplementary Material 1

## Data Availability

The datasets generated and/or analysed during the current study are available from the corresponding author on reasonable request.

## Abbreviations

ACS: Acute coronary syndrome
CGM: Continuous glucose monitoring
CONGA-24: Continuous overall net glycaemic action over 24 h
COPD: Chronic obstructive pulmonary disease
CV: Coefficient of variation
CKD: Chronic kidney disease
DM: Diabetes mellitus
GMI: Glucose management indicator
GV: Glycaemic variability
HBGI: High blood glucose index
LBGI: Low blood glucose index
LVEF: Left ventricular ejection fraction
MAGE: Mean amplitude of glycaemic excursions
MI: Myocardial infarction
SD: Standard deviation
TAR: Time above range 180 mg/dl → 10 mmol/L
TBR: Time below range 70 mg/dl → 3.9 mmol/L
TIR: Time in range 70–180 mg/dL → 3.9–10 mmol/L
TITR: Time in tight range TITR, 70–140 mg/dL → 3.9–7.8 mmol/L
