# Supplementary material for: Continuous glucose monitoring trajectories in patients with acute coronary syndrome
Source: Cardiovasc Diabetol. 2026 Apr 20;25:167. doi: 10.1186/s12933-026-03169-1 (PMC13224573; doi:10.1186/s12933-026-03169-1)

**SUPPLEMENTARY APPENDIX:**

***Continuous Glucose Monitoring Trajectories in Patients with Acute Coronary Syndrome****Diaz-Exposito A et al.*

**Supplementary Table 1. Baseline characteristics of the study population**

| Characteristic | Total (n = 213) |
| --- | --- |
| Demographics |  |
| Age, years | 66.34 ± 11.59 |
| Male sex, n (%) | 166 (77.93%) |
|  |  |
| Cardiovascular risk factors |  |
| Hypertension, n (%) | 149 (69.9%) |
| Diabetes mellitus, n (%) | 94 (44.1%) |
| Type 1 diabetes, n (%) | 2 (0.9%) |
| Type 2 diabetes, n (%) | 93 (43.6%) |
| Hypercholesterolaemia, n (%) | 152 (71.3%) |
| Current smoker, n (%) | 54 (25.3%) |
| Former smoker, n (%) | 98 (46.0%) |
| BMI | 28.41 ± 4.04 kg/m² |
| Normal weight | 35 (16.4%) |
| Overweight | 86 (40.3%) |
| Obesity | 43 (20.1%) |
|  |  |
| Comorbidities |  |
| Atrial fibrillation, n (%) | 10 (4.6%) |
| Previous MI, n (%) | 54 (25.3%) |
| Ischaemic stroke / transient ischaemic attack, n (%) | 12 (5.6%) |
| Peripheral artery disease, n (%) | 17 (7.9%) |
| Heart failure, n (%) | 14 (6.5%) |
| Chronic obstructive pulmonary disease, n (%) | 17 (7.9%) |
| Obstructive sleep apnoea, n (%) | 27 (12.6%) |
| Chronic kidney disease >3 stage, n (%) | 56 (26.2%) |
| Liver disease, n (%) | 2 (0.9%) |
| Family history, n (%) | 134 (62.9%) |
|  |  |
| Medical therapy at discharge |  |
| Statins, n (%) | 209 (98.1%) |
| Ezetimibe, n (%) | 129 (60.5%) |
| PCSK9 inhibitor, n (%) | 5 (2.3%) |
| Fibrates, n (%) | 1 (0.4%) |
| ACE inhibitor, n (%) | 91 (42.7%) |
| Angiotensin II receptor blocker, n (%) | 89 (41.7%) |
| Beta-blocker, n (%) | 170 (79.8%) |
| Mineralocorticoid receptor antagonist, n (%) | 47 (22%) |
| SGLT2 inhibitor, n (%) | 102 (47.8%) |
| GLP-1 receptor agonist, n (%) | 23 (10.8%) |
| Insulin, n (%) | 22 (10.3%) |
| Other antidiabetic agents, n (%) | 81 (38%) |
| Diuretics, n (%) | 69 (32.3%) |
| Systemic corticosteroids, n (%) | 2 (0.9%) |
| Aspirin (ASA), n (%) | 209 (98.1%) |
| Clopidogrel, n (%) | 50 (23.4%) |
| Ticagrelor, n (%) | 112 (52.5%) |
| Prasugrel, n (%) | 50 (23.4%) |
| Non–vitamin K oral anticoagulant (NOAC), n (%) | 22 (10.3%) |
| VKA, n (%) | 1 (0.4%) |
|  |  |
| Medical therapy at 4-months follow-up |  |
| Statins, n (%) | 209 (98.1%) |
| Ezetimibe, n (%) | 138 (64.7%) |
| PCSK9 inhibitor, n (%) | 8 (3.7%) |
| Fibrates, n (%) | 1 (0.4%) |
| ACE inhibitor, n (%) | 84 (39.4%) |
| Angiotensin II receptor blocker, n (%) | 86 (40.3%) |
| Beta-blocker, n (%) | 166 (77.9%) |
| Mineralocorticoid receptor antagonist, n (%) | 44 (20.6%) |
| SGLT2 inhibitor, n (%) | 103 (48.3%) |
| GLP-1 receptor agonist, n (%) | 23 (10.8%) |
| Insulin, n (%) | 21 (9.8%) |
| Other antidiabetic agents, n (%) | 81 (38%) |
| Diuretics, n (%) | 65 (30.5%) |
| Systemic corticosteroids, n (%) | 2 (0.9%) |
| Aspirin (ASA), n (%) | 197 (92.4%) |
| Clopidogrel, n (%) | 58 (27.2%) |
| Ticagrelor, n (%) | 100 (46.9%) |
| Prasugrel, n (%) | 53 (24.8%) |
| Non–vitamin K oral anticoagulant (NOAC), n (%) | 24 (11.2%) |
| VKA, n (%) | 0 (0.00%) |
| ACE, Angiotensin-Converting Enzyme; ASA, Acetylsalicylic Acid; BMI, Body Mass Index; GLP-1, Glucagon-Like Peptide-1; MI, Myocardial Infarction; NOAC, Non–Vitamin K Oral Anticoagulant; PCSK9, Proprotein Convertase Subtilisin/Kexin Type 9; SGLT2, Sodium–Glucose Cotransporter 2; VKA, Vitamin K Antagonist. | |

**Supplementary Table 2: CGM metrics change between baseline and 4-months follow-up according to diabetes status.**

| Metric | Without diabetes summary | Baseline | 4 months | Mean difference (95% CI) | t-test p-value | Wilcoxon p-value | Diabetes summary | Baseline | 4 months | Mean difference (95% CI) | t-test p-value | Wilcoxon p-value |
| --- | --- | --- | --- | --- | --- | --- | --- | --- | --- | --- | --- | --- |
| TIR 70-180 (%) | Median [IQR] | 98.72 [96.85–99.44] | 98.11 [96.49–99.46] | — | — | 0.4724 | Median [IQR] | 85.29 [59.04–93.28] | 80.00 [58.50–93.00] | — | — | 0.3861 |
| TITR 70-140 (%) | Median [IQR] | 91.43 [85.31–95.01] | 88.38 [81.62–93.30] | — | — | 0.0333 | Mean ± SD | 52.58 ± 29.81 | 47.69 ± 27.93 | -4.89 [-10.39, 0.61] | 0.0799 | — |
| TAR >180 (%) | Median [IQR] | 0.43 [0.00–1.04] | 0.48 [0.00–1.54] | — | — | 0.1894 | Median [IQR] | 12.03 [5.01–40.91] | 19.31 [6.43–36.56] | — | — | 0.2081 |
| TBR <70 (%) | Median [IQR] | 0.45 [0.05–1.62] | 0.46 [0.13–2.39] | — | — | 0.6958 | Median [IQR] | 0.23 [0.00–2.01] | 0.08 [0.00–0.58] | — | — | 0.0209 |
| TBR level 2 (<54, %) | Median [IQR] | 0.00 [0.00–0.19] | 0.00 [0.00–0.14] | — | — | 0.9464 | Median [IQR] | 0.00 [0.00–0.18] | 0.00 [0.00–0.03] | — | — | 0.1137 |
| TAR level 2 (>=250, %) | Median [IQR] | 0.00 [0.00–0.00] | 0.00 [0.00–0.00] | — | — | 0.9165 | Median [IQR] | 0.60 [0.00–9.59] | 0.85 [0.02–5.87] | — | — | 0.6154 |
| Mean glucose (mg/dL) | Mean ± SD | 109.30 ± 9.93 | 111.58 ± 11.05 | 2.28 [-0.32, 4.87] | 0.0845 | — | Mean ± SD | 148.71 ± 38.07 | 154.64 ± 42.03 | 5.93 [-2.92, 14.78] | 0.1840 | — |
| SD (mg/dL) | Mean ± SD | 20.26 ± 6.32 | 21.45 ± 6.42 | 1.18 [0.04, 2.33] | 0.0427 | — | Mean ± SD | 38.19 ± 14.74 | 39.31 ± 15.11 | 1.13 [-2.28, 4.54] | 0.5096 | — |
| CV (%) | Mean ± SD | 18.43 ± 4.90 | 19.19 ± 5.16 | 0.76 [-0.33, 1.85] | 0.1695 | — | Median [IQR] | 23.68 [20.40–27.88] | 23.45 [20.53–29.01] | — | — | 0.9207 |
| Intraday CV (%) | Median [IQR] | 15.76 [13.77–18.34] | 16.61 [14.23–20.42] | — | — | 0.0146 | Mean ± SD | 20.63 ± 4.29 | 21.66 ± 4.76 | 1.03 [-0.17, 2.23] | 0.0909 | — |
| GMI (%) | Mean ± SD | 5.92 ± 0.24 | 5.98 ± 0.26 | 0.05 [-0.01, 0.12] | 0.0845 | — | Mean ± SD | 6.87 ± 0.91 | 7.01 ± 1.01 | 0.14 [-0.07, 0.35] | 0.1840 | — |
| MAGE (mg/dL) | Mean ± SD | 40.53 ± 12.94 | 43.38 ± 13.25 | 2.84 [0.93, 4.76] | 0.0043 | — | Mean ± SD | 70.31 ± 23.71 | 74.32 ± 27.19 | 4.01 [-1.83, 9.85] | 0.1735 | — |
| LBGI | Median [IQR] | 0.73 [0.41–1.22] | 0.71 [0.34–1.17] | — | — | 0.6162 | Median [IQR] | 0.19 [0.04–0.84] | 0.13 [0.02–0.44] | — | — | 0.2082 |
| HBGI | Median [IQR] | 0.36 [0.20–0.71] | 0.50 [0.26–0.95] | — | — | 0.0328 | Median [IQR] | 3.02 [1.46–8.32] | 4.18 [1.67–7.03] | — | — | 0.2491 |
| CONGA-24 (mg/dL) | Mean ± SD | 18.84 ± 6.22 | 19.96 ± 5.80 | 1.12 [0.12, 2.13] | 0.0293 | — | Mean ± SD | 32.52 ± 11.42 | 34.95 ± 12.96 | 2.44 [-0.38, 5.25] | 0.0879 | — |
| Hypoglycaemia events (n) | Median [IQR] | 2.00 [0.00–5.00] | 3.00 [1.00–7.75] | — | — | 0.4221 | Median [IQR] | 1.00 [0.00–7.00] | 1.00 [0.00–2.00] | — | — | 0.0194 |
| Hyperglycaemia events (n) | Median [IQR] | 2.00 [0.00–4.00] | 2.50 [0.00–8.00] | — | — | 0.0124 | Mean ± SD | 21.21 ± 13.36 | 25.17 ± 16.35 | 3.96 [-0.11, 8.02] | 0.0561 | — |

Values are presented as mean ± SD or median [IQR], as appropriate for each variable and subgroup. Paired comparisons were performed using the paired t-test for normally distributed variables and the Wilcoxon signed-rank test for non-normally distributed variables.

CONGA-24, Continuous Overall Net Glycemic Action over 24 hours; CV, Coefficient of Variation; GMI, Glucose Management Indicator; HBGI, High Blood Glucose Index; LBGI, Low Blood Glucose Index; MAGE, Mean Amplitude of Glycaemic Excursions; SD, Standard Deviation; TAR, Time Above Range; TBR, Time Below Range; TIR, Time in Range; TTR, Time in Tight Range.

# **Supplementary Table 3**. Baseline CGM-derived metrics at baseline early after an acute coronary syndrome among patients with and without a worsening CGM-trajectory. Worseners were defined as a ΔTITR ≤ −5%. CI:

| Metric | Non-worseners, median [IQR] | Worseners, median [IQR] | p |
| --- | --- | --- | --- |
| Time in range 70–180, % | 97.03 [86.44–99.06] | 95.00 [86.93–98.74] | 0.618 |
| Time in range 70–140, % | 87.64 [69.51–93.28] | 81.59 [59.21–92.49] | 0.438 |
| Time above range >180, % | 0.90 [0.02–9.12] | 2.04 [0.69–10.22] | 0.180 |
| Time below range <70, % | 0.45 [0.00–1.82] | 0.25 [0.00–1.84] | 0.610 |
| Time <54, % | 0.00 [0.00–0.18] | 0.00 [0.00–0.18] | 0.534 |
| Time ≥250, % | 0.00 [0.00–0.46] | 0.00 [0.00–0.56] | 0.735 |
| Mean glucose, mg/dL | 113.54 [105.83–127.56] | 116.21 [108.93–136.61] | 0.362 |
| Standard Deviation | 21.93 [16.81–33.69] | 25.49 [18.66–35.41] | 0.306 |
| Coefficient of variation, % | 19.09 [16.30–24.78] | 20.45 [18.28–25.35] | 0.243 |
| Intraday CV | 17.45 [14.43–20.79] | 18.33 [15.94–20.72] | 0.424 |
| Glucose management indicator, % | 6.03 [5.84–6.36] | 6.09 [5.92–6.58] | 0.362 |
| MAGE, mg/dL | 44.94 [33.68–63.87] | 51.53 [38.80–65.55] | 0.243 |
| LBGI | 0.57 [0.21–0.99] | 0.54 [0.15–1.09] | 0.760 |
| HBGI | 0.62 [0.25–2.11] | 0.94 [0.38–2.54] | 0.286 |
| CONGA-24, mg/dL | 21.37 [15.71–30.04] | 23.95 [17.37–29.48] | 0.420 |
| Hypoglycaemic events, n | 2.00 [0.00–5.50] | 1.00 [0.00–7.25] | 0.441 |
| Hyperglycaemic events, n | 3.00 [0.00–18.50] | 7.50 [2.25–17.50] | 0.192 |

All variables are presented as median [IQR], as all were classified as non-normally distributed according to the Anderson–Darling normality test.

DM, diabetes mellitus. MAGE, mean amplitude of glycaemic excursions; LBGI, low blood glucose index; HBGI, high blood glucose index; CONGA-24, continuous overall net glycaemic action over 24 h; LVEF, left ventricular ejection fraction; MI, myocardial infarction.

**Suplementary Figure 1:** Heatmap presenting averaged time-in-range (A), time-above range (B) and time-in-tight range (C) within daily hours and weekdays in patients without and with diabetes mellitus at baseline. Red color represent higher, while blue color represent lower % of time in and above range. TAR: Time above range; TIR: Time in range; TITR: Time in tight range.

**
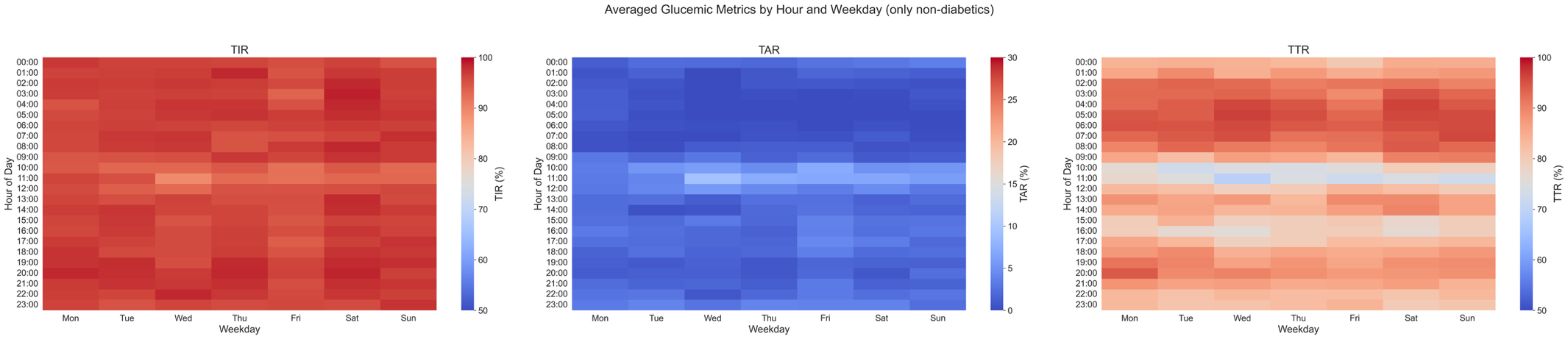
**

TITR

**
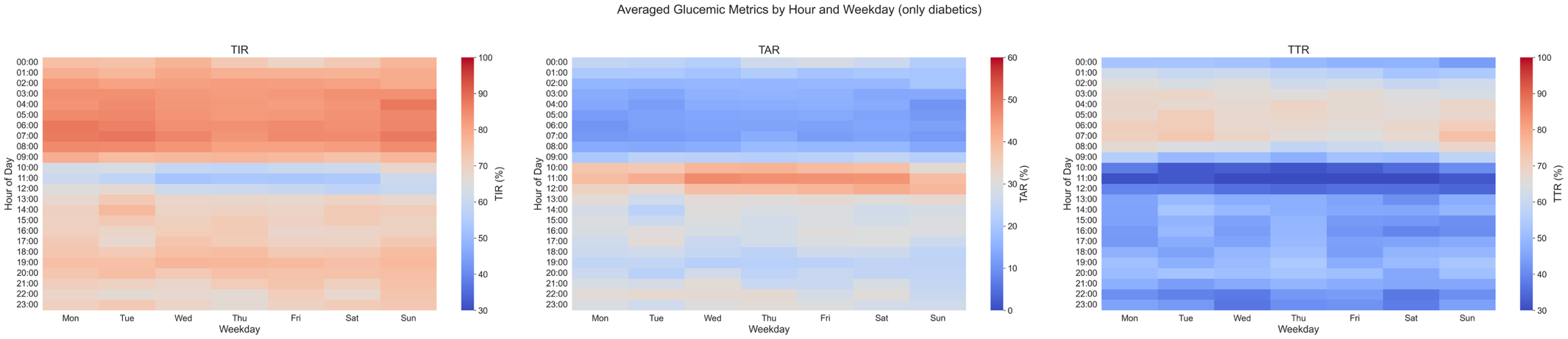
**

TITR

**Supplementary Figure 2:** Heatmap presenting averaged time-in-range (A), time-above range (B) and time-in.tight range (C) within daily hours and weekdays at second visit. Red color represent higher, while blue color represent lower % of time in and above range. TAR: Time above range; TIR: Time in range; TITR: Time in tight range.

**
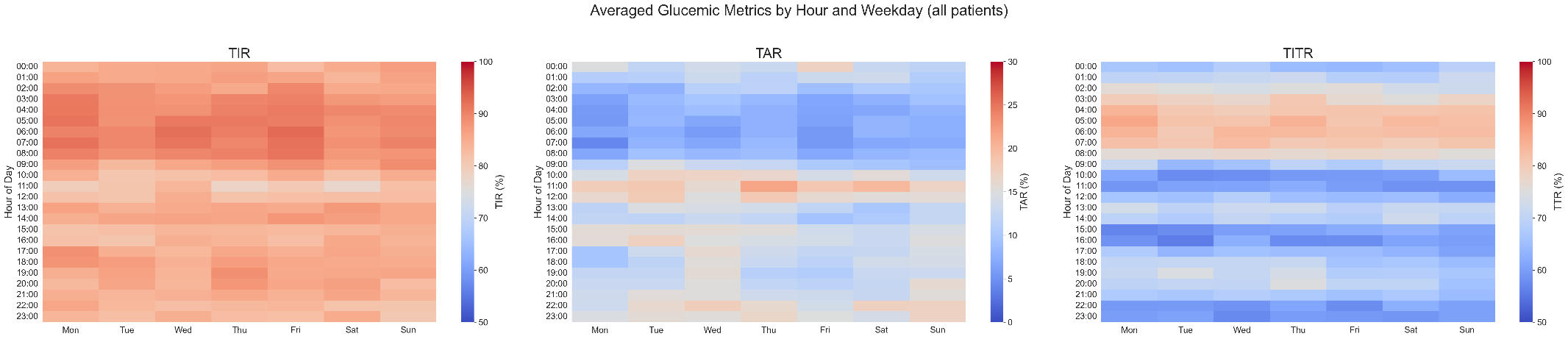
**

**Suplementary Figure 3:** Multivariable model for clinical and glucometric predictors of time-in-tight-range worsening trajectories between baseline and 4-months follow-up. Worseners were defined as a ΔTITR ≤ −5%. CI: Confidence Interval; CKD: Chronic Kidney Disease; CONGA-24: Continuous Overall Net Glycemic Action-24 hour; COPD: Chronic Obstructive Pulmonary Disease; CV: Coefficient of Variation; MAGE: Mean Amplitude of Glucose Excursions; OR: Odds Ratio.


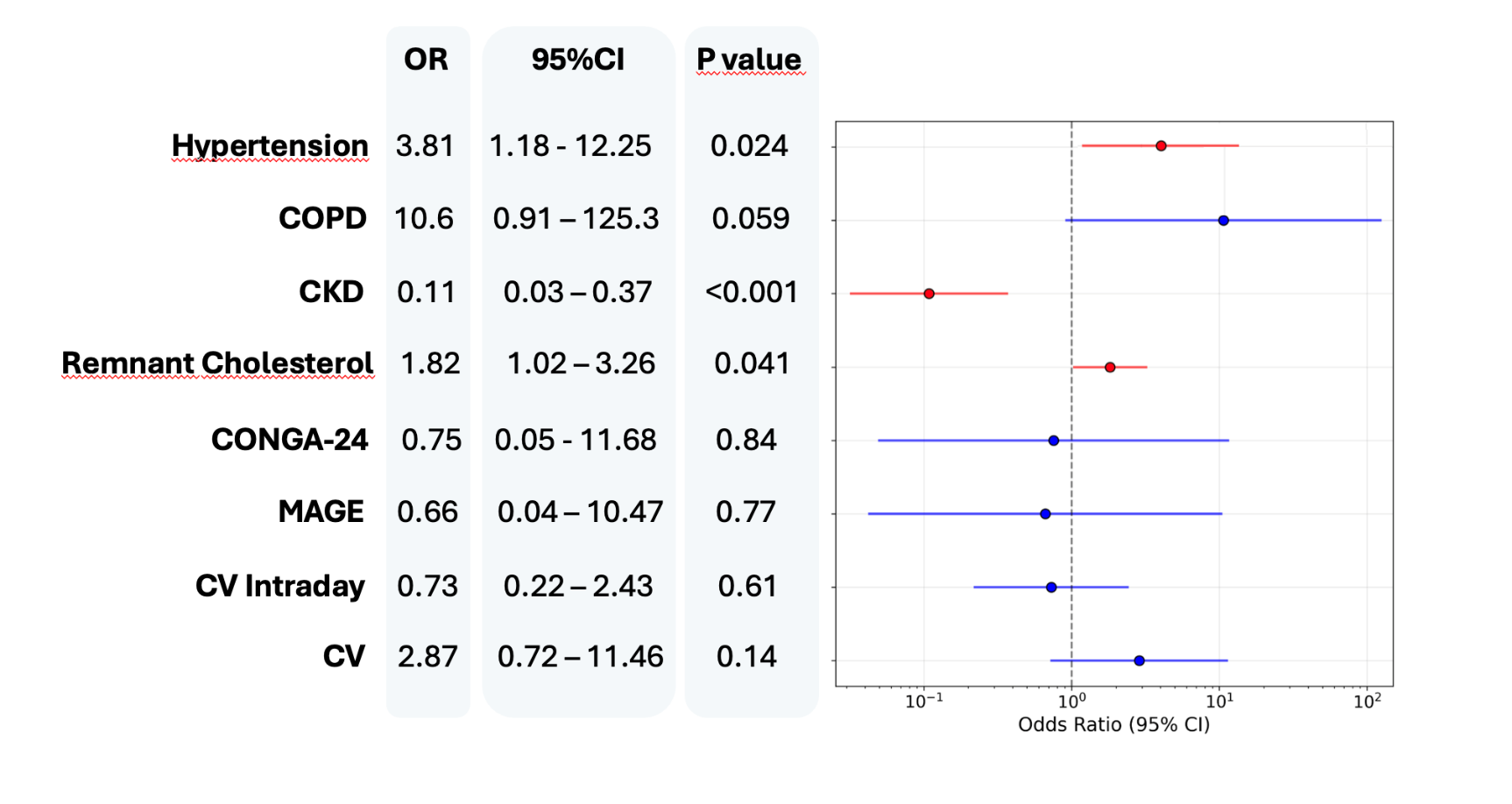

Supplement: Supplementary file 1 — Supplementary Material 1 [file 12933_2026_3169_MOESM1_ESM.docx]
